# Risk of opioid overdose during buprenorphine treatment for opioid use disorder in the fentanyl era

**DOI:** 10.1016/j.addbeh.2026.108603

**Published:** 2026-01-06

**Authors:** Laura C. Chambers, Benjamin D. Hallowell, Andrew R. Zullo, McClaren Rodriguez, Marzan A. Khan, Justin Berk, Rachel Gaither, Macy Daly, Rachel S. Wightman, Francesca L. Beaudoin

**Affiliations:** a Department of Epidemiology, Brown University, Providence, RI, USA; b Substance Use Epidemiology Program, Rhode Island Department of Health, Providence, RI, USA; c Department of Health Services, Policy, and Practice, Brown University, Providence, RI, USA; d Department of Medicine, Alpert Medical School of Brown University, Providence, RI, USA; e Research, Data Evaluation, and Compliance Unit, Rhode Island Department of Behavioral Healthcare, Developmental Disabilities, and Hospitals, Cranston, RI, USA; f Department of Emergency Medicine, Alpert Medical School of Brown University, Providence, RI, USA

**Keywords:** Opioid use disorder, Medications for opioid use disorder, Buprenorphine, Overdose, Opioid overdose, Fentanyl

## Abstract

**Background:**

For patients who use fentanyl, higher than currently recommended maintenance doses of buprenorphine treatment for opioid use disorder (OUD) may be needed to prevent cravings and withdrawal, but some clinicians and regulators are concerned that higher doses may increase overdose risk. We evaluated buprenorphine effectiveness for overdose prevention in the fentanyl era.

**Methods:**

We conducted a retrospective cohort study of Rhode Island residents initiating buprenorphine for OUD (October 2016‒September 2022) using statewide administrative data. On each of 365 follow-up days, patients were classified as having an active buprenorphine prescription (yes/no) and a non-fatal or fatal opioid overdose (yes/no). Follow-up was discontinued if patients died or initiated methadone or naltrexone. Generalized estimating equations compared opioid overdose risk for days with versus without an active buprenorphine prescription, controlling for potential confounders and clustering by patient.

**Results:**

Among 8,676 patients initiating buprenorphine, most were aged 25–44 years (56.0 %) and male (61.3 %). In the 365 days following initiation, 52.6 % of person-days were covered by an active buprenorphine prescription, 1,069 patients (12.3 %) had follow-up discontinued due to methadone initiation, and 411 patients (4.7 %) experienced 545 opioid overdoses. Opioid overdose risk was 61 % lower for days with versus without an active buprenorphine prescription (adjusted risk ratio = 0.39, 95 % confidence interval = 0.31–0.49). Daily doses prescribed on days with and without an opioid overdose event were similar (P = 0.261).

**Conclusions:**

Buprenorphine remains effective for overdose prevention in the fentanyl era among patients who remain in treatment. There was no evidence that higher doses were associated with greater overdose risk.

## 1. Introduction

The United States’ unregulated drug market has become increasingly deadly with the introduction of fentanyl and fentanyl analogues, as well as rising rates of polysubstance use (United States Drug Enforcement Administration, 2021; Friedman and Shover, 2023). Buprenorphine is an effective treatment for opioid use disorder (OUD), with numerous benefits including improved quality of life and reduced risk of overdose and death (Morgan et al., 2019; Sun et al., 2022; Mattick et al., 2014; Sordo et al., 2017; Larochelle et al., 2018; Wakeman et al., 2020; Domzaridou et al., 2022; Xu et al., 2021; Dai et al., 2024). However, widespread fentanyl use has created challenges with buprenorphine treatment initiation and maintenance. Fentanyl is a more efficacious agonist at the mu opioid receptor than morphine, causing greater downregulation of opioid receptor expression, which may lead to higher levels of tolerance (Gillis et al., 2020; McPherson et al., 2010). Precipitated withdrawal on initiation and inadequate control of withdrawal symptoms and cravings have led to concerns about the effectiveness of buprenorphine for patients with chronic fentanyl use (Bisaga, 2019; Silverstein et al., 2019; Danilewitz and McLean, 2020; Jain et al., 2023).

In response, some providers have developed alternate treatment initiation approaches and moved to using higher maintenance doses of buprenorphine (Danilewitz and McLean, 2020; Klaire et al., 2019; Monteiro and Golden, 2021; De Aquino et al., 2020; Hämmig et al., 2016; Carroll et al., 2021; Herring et al., 2021; Baca-Atlas and Williams, 2021). Observational studies suggest that higher buprenorphine maintenance doses are associated with improved treatment retention and reduced acute care utilization in patients with a history of fentanyl use (Chambers et al., 2023; Axeen et al., 2024; Stringfellow et al., 2025). However, despite the well-documented safety profile of buprenorphine, a partial agonist with a ceiling on opioid effects including respiratory depression (Moss et al., 2022; Soyka, 2015; Bell et al., 2009), and increasing clinical data demonstrating the safety and effectiveness of higher buprenorphine doses (Chambers et al., 2023; Axeen et al., 2024; Stringfellow et al., 2025; Grande et al., 2023; Pizzicato et al., 2020; Hser et al., 2014; Kakko et al., 2007; Johnson et al., 2003; Leonardi et al., 2008; United States Food and Drug Administration, 2009; Fareed et al., 2012; Daitch et al., 2014), some clinicians and regulators have voiced concerns that higher doses may increase overdose risk or diversion (Winograd et al., 2023; Textor et al., 2022).

To characterize the effectiveness of buprenorphine treatment for overdose prevention in the fentanyl era, we compared day-level overdose risk for patients with versus without an active buprenorphine prescription covering that day during a period with widespread fentanyl use. Further, to add to the growing body of literature on buprenorphine effectiveness across maintenance doses and inform balanced treatment guidelines, we explored the potential relationship between buprenorphine dose and overdose risk and characterized the substances prescribed and contributing to death for patients with a fatal opioid overdose. Prior studies of buprenorphine treatment outcomes typically summarized buprenorphine treatment engagement over a block period of time, and definitions of treatment engagement often allow for breaks in prescribed buprenorphine of seven days to months (Axeen et al., 2024; Dekker et al., 2025; Bailey et al., 2021; Samples et al., 2020). Additionally, while previous studies have described buprenorphine detection on post-mortem toxicology in the context of overall overdose fatalities or reported overdose death rates among people engaged in buprenorphine treatment, researchers have not directly linked these data (Sordo et al., 2017).

## 2. Methods

### 2.1. Study design and sample

We conducted a retrospective cohort study among adult Rhode Island (RI) residents newly initiating buprenorphine OUD treatment between October 1, 2016, and September 30, 2022. During this period, the vast majority of overdoses in RI involved fentanyl (King et al., 2022). Patients were defined as newly initiating buprenorphine if they did not fill any buprenorphine prescriptions during a six-month washout period (April 1, 2016 – September 30, 2016) and subsequently filled a buprenorphine prescription for the first time during the study period. We followed each patient for up to 365 days after buprenorphine initiation to evaluate ongoing buprenorphine treatment engagement and risk of non-fatal and fatal opioid overdose. The study was approved by the Brown University and RI Department of Health institutional review boards.

### 2.2. Data sources

The study used linked, statewide, individual-level data from the RI Department of Health and RI Department of Behavioral Healthcare, Developmental Disabilities, and Hospitals. Data from the following state databases were included: Prescription Drug Monitoring Program (PDMP), RI Emergency Medical Services (EMS) Information System (RI-EMSIS), Office of the State Medical Examiners (OSME), Center for Vital Records (VR), and Behavioral Health Online Database (BHOLD). We linked the datasets deterministically using name and date of birth.

### 2.3. Measures

#### 2.3.1. Exposure

The study exposure was an active buprenorphine OUD prescription (yes/no) on each day of the 365-day follow-up period, defined using prescription fill data from the PDMP. Buprenorphine products that are not approved by the United States Food and Drug Administration for pain management were considered OUD treatment. We were unable to determine whether patients were experiencing withdrawal symptoms at the time of the initial buprenorphine prescription. An active buprenorphine prescription on a given day was defined as having days’ supply on-hand, based on the prescription fill dates and days’ supply. Study follow-up was discontinued early if/when patients moved out-of-state, started methadone or naltrexone treatment, or experienced a non-overdose death. Patients with a controlled substance prescription fill at a pharmacy outside of RI, Connecticut, and Massachusetts, per PDMP data, were considered to have moved out-of-state. Initiation of methadone was identified using BHOLD records of methadone treatment at an opioid treatment program (OTP). Initiation of naltrexone was identified using prescription fills in the PDMP. Non-overdose deaths were identified using VR death certificate data.

#### 2.3.2. Outcome

The study outcome was non-fatal or fatal opioid overdose (yes/no) on each day of the 365-day follow-up period, defined using RI-EMSIS data on EMS runs for suspected non-fatal opioid overdose and OSME data on unintentional fatal opioid-involved overdoses occurring in RI. Study follow up was continued after non-fatal overdoses. Thus, a patient could experience the study outcome (opioid overdose) on multiple occasions. For patients with a non-fatal and fatal opioid overdose recorded on the same day, only the fatal overdose was included.

#### 2.3.3. Covariates

We selected covariates that we thought were potential confounders *a priori* based on their hypothesized influence on daily buprenorphine engagement and overdose risk. Baseline covariates included age group, sex assigned at birth, distance from home to pharmacy (per ZIP code centroids), and year of treatment initiation, each defined using the patient’s first buprenorphine prescription. Time-varying covariates included an active opioid prescription other than buprenorphine (yes/no) and an active benzodiazepine prescription (yes/no) on each day of the 365-day follow-up period, defined using PDMP data.

#### 2.3.4. Other measures

Other measures considered in this study included baseline health insurance status based on the first buprenorphine prescription, as well as opioid overdose history in the 180 days before treatment initiation based on RI-EMSIS data. For days covered by an active buprenorphine prescription, the total daily dose was determined using fill dates, total quantity dispensed, and days’ supply dispensed on all buprenorphine prescriptions that were active on that day. We categorized daily dose to account for slight variation in prescribing. Patients with active prescriptions for injectable or other alternative buprenorphine formulations on a given day were excluded from daily dose calculations due to the distinctive dosing practices. For non-fatal and fatal opioid overdose events, we summarized whether the patient had active prescriptions for buprenorphine, an opioid other than buprenorphine, and a benzodiazepine on the day of the overdose. For fatal opioid overdose events, we summarized active prescriptions on the day of death and the substances contributing to the cause of death using the OSME determinations, which are based on the totality of evidence available from the death investigation.

### 2.4. Statistical analyses

We used chi-square tests to compare the baseline characteristics of patients with and without a non-fatal or fatal opioid overdose during follow-up. We used generalized estimating equations (GEEs) to compare the risk of non-fatal or fatal opioid overdose for days with versus without an active buprenorphine prescription, controlling for baseline covariates with stabilized inverse probability of treatment weights, controlling for time-varying covariates with traditional adjustment, and clustering by patient. GEEs were specified with a binomial error distribution, log link, exchangeable working correlation, and robust standard errors. Inverse probability of treatment weights were calculated based on baseline age group, sex assigned at birth, distance from home to pharmacy, and year of treatment initiation. As a sensitivity analysis, we fit the GEEs as specified above but controlling for baseline and time-varying covariates with traditional adjustment. To evaluate the potential association between daily buprenorphine dose and opioid overdose risk, we used a chi-squared test to compare the daily dose distribution for days with versus without an overdose event, among days covered by an active buprenorphine prescription. Finally, we compared the baseline characteristics of patients with and without an active buprenorphine prescription on the day of overdose, among patients with a non-fatal opioid overdose only (chi-square tests) and patients with a fatal opioid overdose (Fisher’s exact tests).

## 3. Results

### 3.1. Sample characteristics

Overall, 8,676 adult RI residents initiated buprenorphine OUD treatment between October 1, 2016, and September 30, 2022. The largest number of patients were age 25 to 44 years (55.9 %, n = 4,854) and male (61.3 %, n = 5,319), had private health insurance (43.1 %, n = 3,742), and lived < 5 miles from their pharmacy (73.2 %, n = 6,351) (Table 1). Few patients (4.6 %, n = 395) had a non-fatal opioid overdose in the 180 days before treatment initiation.

### 3.2. Study follow-up

These 8,676 patients contributed 2,886,403 total days of study follow-up (median = 365 days per patient, interquartile range [IQR] = 365–365, range = 2–365). Study follow up was stopped early for several patients: 1,069 (12.3 %) initiated methadone treatment (median = 127 days of follow up, IQR = 54–225), 84 participants (1.0 %) initiated naltrexone treatment (median = 148 days of follow-up, IQR = 80–238), and 67 (0.8 %) died from another cause (median = 158 days of follow-up, IQR = 46–309). No patients met criteria for a move out-of-state during follow-up.

### 3.3. Buprenorphine prescribing

Overall, 1,391 patients (16.0 %) filled one buprenorphine prescription during follow-up, while 7,285 (84.0 %) filled multiple buprenorphine prescriptions. Patients had active buprenorphine prescriptions on 1,517,120 total follow-up days (52.6 %), of which 1.2 % (n = 33,855) were covered by an injectable or other alternative formulation and, thus, excluded from daily dose calculations. Among the remaining 1,483,265 days with an active buprenorphine prescription, the daily doses were: <12 mg (20.2 %, n = 300,155 days), 12 mg (7.6 %, n = 112,625), 16 mg (36.9 %, n = 547,548), 20 mg (7.2 %, n = 106,985), 24 mg (24.8 %, n = 367,225), and ≥ 28 mg (3.3 %, n = 48,727).

### 3.4. Opioid overdose frequency and timing

Overall, 411 patients (4.7 %) experienced 545 non-fatal or fatal opioid overdoses during follow-up. Of 67 patients (0.8 %) who experienced a fatal opioid overdose, 11 (16.4 %) had at least one non-fatal opioid overdose between treatment initiation and their date of death. Overall, 9.3 non-fatal or fatal opioid overdoses occurred per 100,000 person-days covered by an active buprenorphine prescription (141 / 1,517,120), compared to 29.5 per 100,000 person-days without an active buprenorphine prescription (404 / 1,369,283).

Among patients with a non-fatal opioid overdose, the median time to first non-fatal opioid overdose was 138 days (IQR = 56–240, range = 2–364), and the median number of non-fatal opioid overdoses was one (IQR = 1–1, range = 1–9). The median time to fatal opioid overdose was 172 days (IQR = 77–257, range = 2–365).

### 3.5. Characteristics of patients with an opioid overdose

The baseline characteristics of patients with versus without a non-fatal or fatal opioid overdose in the 365 days following treatment initiation were similar, except patients who experienced an overdose were younger (P < 0.001), more likely to have had an overdose in the 180 days before treatment initiation (P < 0.001), and more likely to have Medicaid and less likely to have Medicare insurance (P = 0.026) (Table 1). These differences were most pronounced for patients who experienced a non-fatal opioid overdose only.

The baseline characteristics of patients with versus without an active buprenorphine prescription on the day of the overdose were also similar, both among patients with a non-fatal opioid overdose only and patients with a fatal opioid overdose, with two exceptions (Supplemental Table S1 and S2). Among patients with a non-fatal opioid overdose only, those without an active buprenorphine prescription on the day of the overdose were younger than those with an active buprenorphine prescription (P = 0.019). Among patients with a fatal opioid overdose, those with an active buprenorphine prescription on the day of the overdose were more likely to have had an opioid overdose in the 180 days before treatment initiation than those without an active buprenorphine prescription (P = 0.015).

### 3.6. Association between buprenorphine prescribing and opioid overdose

In GEE analyses, compared to days without an active buprenorphine prescription, patients had a lower risk of non-fatal or fatal opioid overdose on days with an active buprenorphine prescription, controlling for baseline covariates with inverse probability of treatment weights and adjusting for time-varying covariates (risk ratio = 0.39, 95 % confidence interval = 0.31–0.49) (Table 2). This estimate was similar in the sensitivity analysis controlling for both baseline and time-varying covariates with traditional adjustment.

Overall, 348 of 478 non-fatal opioid overdose events (72.8 %) and 56 of 67 fatal opioid overdose events (83.6 %) occurred on a day the patient did not have an active buprenorphine prescription (Table 3). Among the 141 opioid overdose events (non-fatal and fatal) occurring when the patient had an active buprenorphine prescription, 2.8 % (n = 4) occurred on days covered by an injectable or other alternative formulation and were excluded from daily dose calculations. The daily dose distribution was similar for days with an active buprenorphine prescription on which the patient did versus did not experience an overdose event (P = 0.261) (Supplemental Table S3).

### 3.7. Substances prescribed and contributing to opioid overdose deaths

Among the 67 opioid overdose decedents, major substances contributing to the cause of death included: fentanyl (88.1 %, n = 59), cocaine (46.3 %, n = 31), alcohol (17.9 %, n = 12), buprenorphine (13.4 %, n = 9), benzodiazepines (9.0 %, n = 6), methadone (7.5 %, n = 5), amphetamine (7.5 %, n = 5), and methamphetamine (4.5 %, n = 3). Of the nine fatal opioid overdoses among decedents with buprenorphine listed by the medical examiner as a contributing cause of death, all nine had at least one other substance contributing to death, including fentanyl, tramadol, oxycodone, methadone, alcohol, cocaine, amphetamine, methamphetamine, gabapentin, psychiatric medications, and benzodiazepines. There were four fatal opioid overdoses where buprenorphine was detected on post-mortem toxicology but not classified by the medical examiner as contributing to death; all four involved fentanyl. These four decedents all had an active buprenorphine prescription on the day of death.

There were 11 deaths where the decedent had an active buprenorphine prescription for OUD treatment on the date of death, of which two had buprenorphine listed by the medical examiner as a contributing cause of death (Supplemental Table S4). These two decedents had multiple substances contributing to their cause of death besides buprenorphine, including alcohol, oxycodone, diazepam, trazadone, cocaine, quetiapine, and topiramate.

## 4. Discussion

In this statewide study of patients initiating buprenorphine OUD treatment in the fentanyl era, risk of non-fatal or fatal opioid overdose in the year following treatment initiation was over 60 % lower on days with versus without an active buprenorphine prescription. The percentage of patients prescribed different daily dose regimens was similar for days on which the patients did and did not experience an overdose event. This suggests that buprenorphine OUD treatment, including standard and high doses, remains effective for overdose prevention in the fentanyl era, among patients who remain engaged in treatment.

The protective effect associated with buprenorphine in this study is consistent with prior studies using data from before the predominance of fentanyl that found similarly large reductions in overdose risk with active buprenorphine treatment (34–60 %), as well as a cross-sectional study suggesting buprenorphine is protective specifically against fentanyl-involved overdose death (Morgan et al., 2019; Sun et al., 2022; Xu et al., 2021; Dai et al., 2024). However, long-term buprenorphine treatment engagement remains a challenge for many patients (Chua et al., 2023; Dong et al., 2023). Only half of all study days were covered by a buprenorphine prescription, and nine opioid overdose fatalities were observed among patients with an active buprenorphine prescription but without buprenorphine contributing to the cause of death, suggesting the buprenorphine had not been taken as prescribed on those days. There are few evidence-based interventions documented to improve treatment retention (Chan et al., 2021). Long-acting injectable buprenorphine formulations may be helpful for patients who prefer a weekly or monthly injection over a daily medication, although barriers such as prior authorization requirements and high costs currently limit their use (Nelson and Perrone, 2023). Higher than standard maintenance daily doses of buprenorphine should also be considered for patients using fentanyl to improve treatment retention (Bisaga, 2019; Silverstein et al., 2019; Danilewitz and McLean, 2020; Baca-Atlas and Williams, 2021; Chambers et al., 2023; Axeen et al., 2024; Stringfellow et al., 2025; Grande et al., 2023; Greenwald et al., 2022).

Importantly, over 12 % of study patients transitioned from buprenorphine to methadone. The ability to link to statewide methadone treatment data from OTPs was a strength in this study, as most retrospective studies using PDMP data do not account for methadone switching. In the United States, buprenorphine is available in office-based settings and OTPs, whereas methadone is available for OUD only at OTPs. Some patients may prefer the more structured environment of the OTP; however, it is more likely that patients may have preferred a full agonist (methadone) over a partial agonist (buprenorphine) due to pharmacologic differences. For example, some patients with chronic daily fentanyl use have reported ongoing withdrawal symptoms even at higher doses of buprenorphine (Danilewitz and McLean, 2020; Baca-Atlas and Williams, 2021). Additionally, buprenorphine binds avidly to opioid receptors in a competitive fashion reducing opioid effects of fentanyl if used concurrently (Moss et al., 2022; Volpe et al., 2011). Other reasons for transition to methadone could include increased challenges with buprenorphine initiation in the era of fentanyl, such as higher rates of precipitated withdrawal and/or the need for a longer withdrawal period prior to buprenorphine initiation (Bisaga, 2019; Silverstein et al., 2019; Greenwald et al., 2022; Antoine et al., 2021). Further evaluation of treatment switching (e.g., patient characteristics, reasons for transitions, and outcomes) and comparisons of the effectiveness between buprenorphine and methadone are needed.

In this study, there was no evidence that higher buprenorphine doses were associated with greater overdose risk. We did not have sufficient study power to estimate the causal effects of different dosing strategies for buprenorphine or to identify an optimal dosing strategy, which are important areas for future work and may require the application of innovative methods such as the clone-censor-weight approach (Zanette et al., 2025). Nonetheless, in our study, for opioid overdoses that occurred on days with an active buprenorphine prescription, the daily doses prescribed were similar to those prescribed on days without an overdose event. This suggests that higher buprenorphine doses can be used to improve treatment retention among patients using fentanyl without increasing overdose risk. However, our study and previous studies of higher than standard buprenorphine doses were observational and, thus, may be subject to confounding (e.g., by factors influencing prescribed dose). A sufficiently-powered randomized controlled trial evaluating the effect of higher daily buprenorphine doses on treatment retention, overdose risk, and patient safety is needed to confirm these findings and inform treatment guidelines.

This study had limitations. First, buprenorphine treatment was assessed using dispensed prescriptions, which may not reflect actual buprenorphine use (e.g., patients may not take the buprenorphine as prescribed and/or may use non-prescribed buprenorphine). However, we expect this potential misclassification to bias the association of interest toward the null. Second, our analyses of daily dose excluded long-acting injectable and other alternative formulations due to distinctive dosing practices, so our findings may not be generalizable to patients receiving these formulations. Third, our study was limited to people who initiated buprenorphine treatment, which requires access to and engagement in care. People with an OUD who experience barriers to treatment access may differ in important ways from the people in our study, and such characteristics could impact buprenorphine effectiveness. Fourth, non-fatal opioid overdoses that did not result in call for emergency assistance would not be captured using EMS data, although we expect this to affect people with and without active buprenorphine prescriptions similarly. Fifth, toxicology testing data were not available for non-fatal opioid overdose events. Future research evaluating whether people engaged in buprenorphine treatment had buprenorphine present in their system at the time of non-fatal opioid overdose would be useful to further understand the extent to which buprenorphine protects against and contributes to non-fatal opioid overdose in the fentanyl era. Sixth, as noted above, this study was observational and, thus, may be subject to confounding. The PDMP database does not contain information on some factors that may impact daily buprenorphine treatment engagement, prescribing practices, and overdose risk (e.g., social determinants of health, comorbidities, co-prescribed medications that are not scheduled, and engagement with other services and supports). Additionally, although the PDMP includes information on health insurance status, we did not feel it was appropriate to include this measure in our analysis due to known measurement error. Despite these limitations, this study was strengthened by its statewide population, consideration of both baseline and time-varying measured potential confounders, and ability to account for transitions to other medications for OUD.

In the year following initiation of buprenorphine OUD treatment, non-fatal and fatal opioid overdoses were extremely rare on days when patients had active buprenorphine prescriptions, which reduced opioid overdose risk by over 60 %. Buprenorphine is still effective for overdose prevention in the era of fentanyl, among patients who remain engaged in treatment. Future research to evaluate the risks and benefits of strategies for supporting patients with a history of fentanyl use to remain engaged in buprenorphine OUD treatment is essential given a drug supply dominated by fentanyl.

## Supplementary Material

Published supplement

## Figures and Tables

**Table 1:** Baseline characteristics* of adult Rhode Island residents initiating buprenorphine treatment for OUD (October 2016 – September 2022), overall and stratified by subsequent non-fatal or fatal opioid overdose in the 365 days following treatment initiation.

		Any opioid overdose in the 365 days following treatment initiation			
	Overall N = 8,676 n (%)	No overdose N = 8,265 n (%)	Non-fatal overdose only^†^ N = 344 n (%)	Fatal overdose N = 67 n (%)	P-value
**Age group (years)**					
18–24	655 (7.6)	577 (7.0)	73 (21.2)	5 (7.5)	<0.001
25–34	2,524 (29.1)	2,372 (28.7)	123 (35.8)	29 (43.3)	
35–44	2,330 (26.9)	2,239 (27.1)	74 (21.5)	17 (25.4)	
45–54	1,600 (18.4)	1,546 (18.7)	47 (13.7)	7 (10.5)	
55 or older	1,567 (18.1)	1,531 (18.5)	27 (7.9)	9 (13.4)	
**Sex assigned at birth**					
Female	3,232 (37.3)	3,092 (37.4)	120 (34.9)	20 (29.9)	0.424
Male	5,319 (61.3)	5,054 (61.2)	218 (63.4)	47 (70.2)	
Unknown	125 (1.4)	119 (1.4)	6 (1.7)	0 (0.0)	
**Health insurance status**					
Medicaid	3,050 (35.2)	2,888 (34.9)	138 (40.1)	24 (35.8)	0.026
Medicare	893 (10.3)	869 (10.5)	20 (5.8)	4 (6.0)	
Private	3,742 (43.1)	3,573 (43.2)	142 (41.3)	27 (40.3)	
Other or none	991 (11.4)	935 (11.3)	44 (12.8)	12 (17.9)	
**Distance from home to pharmacy (miles)** ^‡^					
Less than 5	6,351 (73.2)	6,062 (73.4)	242 (70.4)	47 (70.2)	0.375
5 or more	2,293 (26.4)	2,174 (26.3)	99 (28.8)	20 (29.9)	
Unknown	32 (0.4)	29 (0.4)	3 (0.9)	0 (0.0)	
**Opioid overdose in the 180 days prior to treatment initiation**					
Yes	395 (4.6)	320 (3.9)	66 (19.2)	9 (13.4)	<0.001
No	8,281 (95.5)	7,945 (96.1)	278 (80.8)	58 (86.6)	
**Year of treatment initiation**					
2016	549 (6.3)	520 (6.3)	24 (7.0)	5 (7.5)	0.439
2017	2,025 (23.3)	1,923 (23.3)	85 (24.7)	17 (25.4)	
2018	1,800 (20.8)	1,736 (21.0)	53 (15.4)	11 (16.4)	
2019	1,383 (15.9)	1,308 (15.8)	66 (19.2)	9 (13.4)	
2020	1,141 (13.2)	1,084 (13.1)	48 (14.0)	9 (13.4)	
2021	1,042 (12.0)	995 (12.0)	38 (11.1)	9 (13.4)	
2022	736 (8.5)	699 (8.5)	30 (8.7)	7 (10.5)	
**Initial buprenorphine formulation**					
Film	5,231 (60.3)	5,011 (60.6)	186 (54.1)	34 (50.8)	0.065
Tablet	3,440 (39.7)	3,249 (39.3)	158 (45.9)	33 (49.3)	
ER solution	5 (0.1)	5 (0.1)	0 (0.0)	0 (0.0)	

Abbreviations: ER, extended release; OUD, opioid use disorder.

* Defined based on the first buprenorphine prescription.

† Excludes patients with both a non-fatal and fatal opioid overdose during this period.

‡ Based on ZIP-code centroids.

**Table 2:** Analyses comparing the risk of non-fatal or fatal opioid overdose in the 365 days following buprenorphine treatment initiation for days with versus without an active buprenorphine prescription.

	Risk ratio (95 % CI)
**Unadjusted analysis – no control for covariates**	
Days with an active buprenorphine prescription	0.36 (0.29, 0.44)
Days without an active buprenorphine prescription	Ref.
**Primary analysis – IPTWs to control for baseline covariates, adjustment to control for time-varying covariates** *	
Days with an active buprenorphine prescription	0.39 (0.31, 0.49)
Days without an active buprenorphine prescription	Ref.
**Sensitivity analysis – adjustment to control for baseline and time-varying covariates** *	
Days with an active buprenorphine prescription	0.37 (0.30, 0.46)
Days without an active buprenorphine prescription	Ref.

Abbreviations: CI, confidence interval; IPTW, inverse probability of treatment weight.

* Baseline covariates included age group, sex assigned at birth, distance from home to pharmacy, and year of treatment initiation. Time-varying covariates included an active prescription for an opioid other than buprenorphine and an active prescription for a benzodiazepine.

**Table 3:** Select active prescriptions on the day of non-fatal and fatal opioid overdoses.

	Overall opioid overdose events N = 545 n (%)	Non-fatal opioid overdose events N = 478 n (%)	Fatal opioid overdose events N = 67 n (%)
**Active buprenorphine prescription**			
Yes	141 (25.9)	130 (27.2)	11 (16.4)
Daily dose (mg)*			
12 or less	28 (5.1)	26 (5.4)	2 (3.0)
16	56 (10.3)	52 (10.9)	4 (6.0)
20	10 (1.8)	9 (1.9)	1 (1.5)
24	41 (7.5)	39 (8.2)	2 (3.0)
28 or more	2 (0.4)	1 (0.2)	1 (1.5)
No	404 (74.1)	348 (72.8)	56 (83.6)
**Active opioid prescription other than buprenorphine**			
Yes^†^	7 (1.3)	3 (0.6)	4 (6.0)
No	538 (98.7)	475 (99.4)	63 (94.0)
**Active benzodiazepine prescription**			
Yes	42 (7.7)	33 (6.9)	9 (13.4)
No	503 (92.3)	445 (93.1)	58 (86.6)

* Daily dose categorized to account for slight variation in prescribing: 12 mg or less (less than 14), 16 mg (14 to less than 18), 20 mg (18 to less than 22), 24 mg (22 to less than 26), 28 or more mg (26 or more). Patients with an active prescription for an injectable or other alternative buprenorphine formulation were excluded from the daily dose calculation.

† Daily morphine milligram equivalents ranged from 40 to 390.

## Data Availability

The authors do not have permission to share data.
