# Supplementary material for: Risk of opioid overdose during buprenorphine treatment for opioid use disorder in the fentanyl era
Source: Addict Behav. Author manuscript; Available in PMC 2026 Feb 26. (PMC12937454; doi:10.1016/j.addbeh.2026.108603)
Supplement: Published supplement [file NIHMS2144798-supplement-Published_supplement.pdf]

SUPPLEMENTAL MATERIAL

Title: Risk of Opioid Overdose During Buprenorphine Treatment for Opioid Use Disorder in the Fentanyl Era

Investigators: Laura C. Chambers, Benjamin D. Hallowell, Andrew R. Zullo, McClaren Rodriguez, Marzan A. Khan, Justin Berk, Rachel Gaither, Macy Daly, Rachel S. Wightman, Francesca L. Beaudoin

Table of Contents:

- **Table S1**. Baseline characteristics of adult Rhode Island residents initiating buprenorphine treatment for OUD (October 2016 – September 2022), overall and among those with a non-fatal opioid overdose only in the 365 days following treatment initiation stratified by buprenorphine prescription status on the day of their first non-fatal opioid overdose.

- **Table S2**. Baseline characteristics of adult Rhode Island residents initiating buprenorphine treatment for OUD (October 2016 – September 2022), overall and among those with a fatal opioid overdose in the 365 days following treatment initiation stratified by buprenorphine prescription status on the day of fatal opioid overdose.

- **Table S3**. Daily dose distribution for days with an active buprenorphine prescription, overall and stratified by whether the patient experienced a non-fatal or fatal opioid overdose that day, in the 365 days following initiation of buprenorphine treatment for OUD among Rhode Island residents (October 2016 – September 2022).

- **Table S4**. Substances prescribed on the day of death and substances contributing to the cause of death, among adult Rhode Island residents who experienced a fatal opioid overdose in the 365 days following initiation of buprenorphine treatment for OUD (October 2016 – September 2022).

Table S1. Baseline characteristics* of adult Rhode Island residents initiating buprenorphine treatment for OUD (October 2016 – September 2020), overall and among those with a non-fatal opioid overdose only in the 365 days following treatment initiation stratified by buprenorphine prescription status on the day of their first non-fatal opioid overdose.

		**Non-fatal opioid overdose only in the 365 days following treatment initiation†**		
	**Overall**<br>N=8,676<br>n (%)	**Active buprenorphine prescription**<br>N=99<br>n (%)	**No active buprenorphine prescription**<br>N=245<br>n (%)	**P-value**
Age group (years)				
18-24	655 (7.6)	11 (11.1)	62 (25.3)	0.019
25-34	2,524 (29.1)	37 (37.4)	86 (35.1)	
35-44	2,330 (26.9)	29 (29.3)	45 (18.4)	
45-54	1,600 (18.4)	12 (12.1)	35 (14.3)	
55 or older	1,567 (18.1)	10 (10.1)	17 (6.9)	
Sex assigned at birth				
Female	3,232 (37.3)	38 (38.4)	82 (33.5)	0.581
Male	5,319 (61.3)	60 (60.6)	158 (64.5)	
Unknown	125 (1.4)	1 (1.0)	5 (2.0)	
Health insurance status				
Medicaid	3,050 (35.2)	39 (39.4)	99 (40.4)	0.176
Medicare	893 (10.3)	10 (10.1)	10 (4.1)	
Private	3,742 (43.1)	37 (37.4)	105 (42.9)	
Other or none	991 (11.4)	13 (13.1)	31 (12.7)	
Distance from home to pharmacy (miles)‡				
Less than 5	6,351 (73.2)	64 (64.7)	178 (72.7)	0.154
5 or more	2,293 (26.4)	33 (33.3)	66 (26.9)	
Unknown	32 (0.4)	2 (2.0)	1 (0.6)	
Opioid overdose in the 180 days prior to treatment initiation				
Yes	395 (4.6)	14 (14.1)	52 (21.2)	0.131
No	8,281 (95.5)	85 (85.9)	193 (78.8)	
Year of treatment initiation				
2016	549 (6.3)	7 (7.1)	17 (6.9)	0.292
2017	2,025 (23.3)	17 (17.2)	68 (27.8)	
2018	1,800 (20.8)	19 (19.2)	34 (13.9)	
2019	1,383 (15.9)	22 (22.2)	44 (18.0)	
2020	1,141 (13.2)	15 (15.2)	33 (13.5)	
2021	1,042 (12.0)	8 (8.1)	30 (12.2)	
2022	736 (8.5)	11 (11.1)	19 (7.8)	
Initial buprenorphine formulation				
Film	5,231 (60.3)	60 (60.6)	126 (51.4)	0.122
Tablet	3,440 (39.7)	39 (39.4)	119 (48.6)	
ER solution	5 (0.1)	0 (0.0)	0 (0.0)	

Abbreviations: ER, extended release; OUD, opioid use disorder.

* Defined based on the first buprenorphine prescription.
† Excludes patients with both a non-fatal and fatal opioid overdose during this period.
‡ Based on ZIP-code centroids.

Table S2. Baseline characteristics* of adult Rhode Island residents initiating buprenorphine treatment for OUD (October 2016 – September 2022), overall and among those with a fatal opioid overdose in the 365 days following treatment initiation stratified by buprenorphine prescription status on the day of fatal opioid overdose.

		**Fatal opioid overdose in the 365 days following treatment initiation**		
	**Overall** N=8,676 n (%)	**Active buprenorphine prescription** N=11 n (%)	**No active buprenorphine prescription** N=56 n (%)	**P-value**
Age group (years)				
18-24	655 (7.6)	1 (9.1)	4 (7.1)	0.443
25-34	2,524 (29.1)	5 (45.5)	24 (42.9)	
35-44	2,330 (26.9)	1 (9.1)	16 (28.6)	
45-54	1,600 (18.4)	1 (9.1)	6 (10.7)	
55 or older	1,567 (18.1)	3 (27.3)	6 (10.7)	
Sex assigned at birth				
Female	3,232 (37.3)	4 (36.4)	16 (28.6)	0.721
Male	5,319 (61.3)	7 (63.6)	40 (71.4)	
Unknown	125 (1.4)	0 (0.0)	0 (0.0)	
Health insurance status				
Medicaid	3,050 (35.2)	6 (54.6)	18 (32.1)	0.428
Medicare	893 (10.3)	1 (9.1)	3 (5.4)	
Private	3,742 (43.1)	3 (27.3)	24 (42.9)	
Other or none	991 (11.4)	1 (9.1)	11 (19.6)	
Distance from home to pharmacy (miles)†				
Less than 5	6,351 (73.2)	9 (81.8)	38 (67.9)	0.484
5 or more	2,293 (26.4)	2 (18.2)	18 (32.1)	
Unknown	32 (0.4)	0 (0.0)	0 (0.0)	
Opioid overdose in the 180 days prior to treatment initiation				
Yes	395 (4.6)	4 (36.4)	5 (8.9)	0.034
No	8,281 (95.5)	7 (63.6)	51 (91.1)	
Year of treatment initiation				
2016	549 (6.3)	1 (9.1)	4 (7.1)	0.903
2017	2,025 (23.3)	4 (36.4)	13 23.2)	
2018	1,800 (20.8)	2 (18.2)	9 (16.1)	
2019	1,383 (15.9)	1 (9.1)	8 (14.3)	
2020	1,141 (13.2)	1 (9.1)	8 (14.3)	
2021	1,042 (12.0)	2 (18.2)	7 (12.5)	
2022	736 (8.5)	0 (0.0)	7 (12.5)	
Initial buprenorphine formulation				
Film	5,231 (60.3)	6 (54.6)	28 (50.)	0.783
Tablet	3,440 (39.7)	5 (45.5)	28 (50.0)	
ER solution	5 (0.1)	0 (0.0)	0 (0.0)	

Abbreviations: ER, extended release; OUD, opioid use disorder.

* Defined based on the first buprenorphine prescription.

† Based on ZIP-code centroids.

Table S3. Daily dose distribution for days with an active buprenorphine prescription for OUD treatment, overall and stratified by whether the patient experienced a non-fatal or fatal opioid overdose that day, in the 365 days following initiation of buprenorphine treatment for OUD among Rhode Island residents (October 2016 – September 2022).

	**All days with a buprenorphine prescription** N=1,483,265 n (%)	**Opioid overdose event that day**		
		**Yes** N=137 n (%)	**No** N=1,483,128 n (%)	**P-value**
Daily dose (mg)*				
8 or less	300,155 (20.2)	22 (16.1)	300,133 (20.2)	0.261
12	112,625 (7.6)	6 (4.4)	112,619 (7.6)	
16	547,548 (36.9)	56 (40.9)	547,492 (36.9)	
20	106,985 (7.2)	10 (7.3)	106,975 (7.2)	
24	367,225 (24.8)	41 (29.9)	367,184 (24.8)	
28 or more	48,727 (3.3)	2 (1.5)	48,725 (3.3)	

Abbreviations: OUD, opioid use disorder.

* Daily dose categorized to account for slight variation in prescribing: 8 mg or less (less than 10), 12 mg (10 to less than 14), 16 mg (14 to less than 18), 20 mg (18 to less than 22), 24 mg (22 to less than 26), 28 or more mg (26 or more). Patients with an active prescription for an injectable or other alternative buprenorphine formulation were excluded from the daily dose calculation.

Table S4. Substances prescribed on the day of death and substances contributing to the cause of death, among adult Rhode Island residents who experienced a fatal opioid overdose in the 365 days following initiation of buprenorphine treatment for OUD (October 2016 – September 2022).

	**Fatal opioid overdose events** N=67 n (%)	**Fatal opioid overdose events among decedents with an active buprenorphine prescription for OUD treatment** N=11 n (%)	**Fatal opioid overdose events among decedents with buprenorphine as a contributing cause of death** N=9 n (%)
Active prescriptions on the day of death			
Buprenorphine OUD treatment	11 (16.4)	11 (100.0)	2 (22.2)
Daily dose (mg)*			
12 or less	2 (3.0)	2 (18.2)	0 (0.0)
16	4 (6.0)	4 (36.4)	1 (11.1)
20	1 (1.5)	1 (9.1)	0 (0.0)
24	2 (3.0)	2 (18.2)	1 (11.1)
28 or more	1 (2.0)	1 (9.1)	0 (0.0)
Opioid other than buprenorphine OUD treatment	4 (6.0)	1 (9.1)	1 (11.1)
Benzodiazepine	9 (13.4)	1 (9.1)	0 (0.0)
Substances contributing to cause of death			
Opioid	67 (100.0)	11 (100.0)	9 (100.0)
Fentanyl	59 (88.1)	9 (81.8)	5 (55.6)
Buprenorphine	9 (13.4)	2 (18.2)	9 (100.0)
Methadone	5 (7.5)	0 (0.0)	2 (22.2)
Stimulant	35 (52.2)	7 (63.6)	2 (22.2)
Cocaine	31 (46.3)	6 (54.6)	1 (11.1)
Amphetamine	5 (7.5)	1 (9.1)	1 (11.1)
Methamphetamine	3 (4.5)	1 (9.1)	1 (11.1)
Alcohol	12 (17.9)	2 (18.2)	3 (33.3)
Benzodiazepine	6 (9.0)	2 (18.2)	2 (22.2)

* Daily dose categorized to account for slight variation in prescribing: 12 mg or less (less than 14), 16 mg (14 to less than 18), 20 mg (18 to less than 22), 24 mg (22 to less than 26), 28 or more mg (26 or more). Patients with an active prescription for an injectable or other alternative buprenorphine formulation were excluded from the daily dose calculation.
